# Exploring the correlation between genetic transcription and multi-temporal developmental autism spectrum disorder using resting-state functional magnetic resonance imaging

**DOI:** 10.3389/fnins.2023.1219753

**Published:** 2023-06-29

**Authors:** Yanling Li, Fanchao Zhou, Rui Li, Jiahe Gu, Jiangping He

**Affiliations:** School of Electrical Engineering and Electronic Information, Xihua University, Chengdu, China

**Keywords:** autism spectrum disorder, multi-tempora, transcription-neuroimaging association analysis, resting-state functional magnetic resonance imaging, regional homogeneity, amplitude of low frequency fluctuation, fractional amplitude of low-frequency fluctuation, degree centrality

## Abstract

**Introduction:**

The present investigation aimed to explore the neurodevelopmental trajectory of autism spectrum disorder (ASD) by identifying the changes in brain function and gene expression associated with the disorder. Previous studies have indicated that ASD is a highly inherited neurodevelopmental disorder of the brain that displays symptom heterogeneity across different developmental periods. However, the transcriptomic changes underlying these developmental differences remain largely unknown.

**Methods:**

To address this gap in knowledge, our study employed resting-state functional magnetic resonance imaging (rs-fMRI) data from a large sample of male participants across four representative age groups to stratify the abnormal changes in brain function associated with ASD. Partial least square regression (PLSr) was utilized to identify unique changes in gene expression in brain regions characterized by aberrant functioning in ASD.

**Results:**

Our results revealed that ASD exhibits distinctive developmental trajectories in crucial brain regions such as the default mode network (DMN), temporal lobe, and prefrontal lobes during critical periods of neurodevelopment when compared to the control group. These changes were also associated with genes primarily located in synaptic tissues.

**Discussion:**

The findings of this study suggest that the neurobiology of ASD is uniquely heterogeneous across different ages and may be accompanied by distinct molecular mechanisms related to gene expression.

## Introduction

Autism spectrum disorder is a mental illness with distinct genetic characteristics that persist throughout the life cycle. The condition is typified by external manifestations such as impaired social and communicative abilities, intellectual disability, and restricted interests or repetitive behaviors. Existing research indicates that the symptoms of ASD undergo age-dependent changes (Magiati et al., 2014; Lever and Geurts, 2018), yet the underlying pathogenetic mechanisms of these changes have not been uniformly elucidated.

The manifestation of ASD varies with age, and it is generally believed that cognitive ability improves with age and certain symptoms ameliorate throughout the lifespan (Happe et al., 2016). During the period of 6–12 years old, children’s social interaction behavior and understanding of social relationships are the most obvious characteristics of ASD. By the age of 12–15, the condition is relatively stable. Some patients’ symptoms have improved, but some will also have serious degeneration. The gray matter density of the cerebral cortex continues to increase at the age of 15–30, and stops at the age of 30 (Giedd et al., 1999; Sowell et al., 2003; Shaw et al., 2006). Since ASD is a typical neurological disorder of the brain, it is very important and meaningful to use magnetic resonance imaging technology to explore developmental alterations within the pathological regions of the brain. The functional connectivity between the cerebellum and default mode network (DMN) displays greater variations in children with ASD than adolescents (Haghighat et al., 2021), whereas adults with ASD have significantly lower DC in Wernicke’s area compared to children and adolescents (Lee et al., 2017). While the current research on the brain stratification development of ASD lacks unanimity, the abnormal changes of specific cerebral cortexes such as DMN in key age groups have become a consensus (Doyle-Thomas et al., 2015).

Genetic factors play a dominant role in the etiology of autism. Despite this, a limited number of articles have specifically explained changes in genetic transcription in stratified studies of ASD age (Rahbar et al., 2015; Bao et al., 2023). The neuropathological mechanism of autism is complex, involving abnormal synaptic function, abnormal neuronal activity patterns, abnormal genes, an abnormal immune system, etc. (Won et al., 2013; Zhang et al., 2015). For example, inhibitory neurotransmitters, such as gamma-aminobutyric acid (GABA) and glutamate, have been implicated as primary contributors to the onset of autism (Marotta et al., 2020). Increasing evidence has highlighted the biological basis of autism, and indicated that the disorder of excitatory and inhibitory regulation between neurons leads to an ASD signaling imbalance (Culotta and Penzes, 2020).

In this study, we aimed to identify alterations in brain function and gene transcription associated with ASD across four distinct age groups using transcriptomic-neuroimaging spatial association analysis. To achieve this, four metrics, namely, Amplitude of Low-Frequency Fluctuations (ALFF), fractional Amplitude of Low-Frequency Fluctuations (fALFF), Degree Centrality (DC), and Regional Homogeneity (ReHo) were calculated using a large resting-state fMRI dataset, and the resulting heterogeneous changes in ASD brain across four age groups were characterized by t-map, after ComBat Harmonization removal site effect. Finally, partial least squares analysis (PLS) was performed to interpret the weighted linear combination between brain functional activities and the genome, and the enriched significant correlation genes were identified.

## Methods

### Subjects

For the study, we used resting-state fMRI data from autism brain imaging data exchange (ABIDE),^1^ a total of 396 male subjects were enrolled, consisting of 202 ASD (age range 6–39) and 194 TD (typical development, age range 6–56). The way the brain develops with age, both in terms of structure and function, as well as the signs of ASD, varies depending on the age group (Giedd et al., 1999; Sowell et al., 2003; Shaw et al., 2006). The sample was subdivided into four age groups: stage 1 (children, age range 6 ~ 12), stage 2 (adolescents, age range 12 ~ 15), stage 3 (young-adult, age range 15 ~ 30), stage 4 (adult, age range 30 ~ 56). Given the cross-sectional nature of the data， a rigorous screening process was employed to minimize potential experimental errors caused by individual differences. Specifically, subjects were excluded if their head displacement was greater than 2 mm if their average frame displacement exceeded 0.5, or if the ratio of missing volumes exceeded 0.5.

### Preprocessing

The present study utilized the DPARSFA software^2^ to preprocess fMRI data. The preprocessing steps included: 1. Remove the first 10 volumes of individual subjects; 2. Remove the volumes with a maximum head motion greater than 2 mm, and re-align the remaining rolls; 3. Register all fMRI data to the standard EPI template of Montreal Neurological Institute MNI-152 and resampled to a voxel size of 3 × 3 × 3 mm; 4. Remove the linear trends of fMRI data; 5. Calculate the ALFF map of each subject, and use the ratio of ALFF to the root mean square of the power spectrum of all frequencies to calculate the fALFF map, and subsequently normalize Z-transformation; 6. Use bandpass filter (0.01 < *f* < 0.1 Hz) to remove noise signals from the BOLD time series; 7. Estimate nuisance covariates from white matter and cerebrospinal fluid (CSF) and subsequent regression from the resting-state fMRI data along with motion realignment parameters and global mean signal for confounding effects elimination; 8. Use cubic spline interpolation to scrub the volume of image mean frame displacement (FD) > 0.5 mm for the effect of head movement reduction. Finally, the regional homogeneity (ReHo) and degree centrality (DC) maps for each subject were normalized by Z-transform and spatially smoothed using an 8 mm half-height full-width Gaussian kernel.

### Measurements

In our work, DPARSFA (see text footnote 2) was employed to derive several neuroimaging metrics including ALFF, fALFF, DC, and ReHo. ALFF characterizes the intensity of low-frequency oscillations in each voxel by computing the square root of the power spectrum within a certain frequency range. fALFF is divided by the ALFF of each voxel to improve the effect of noise on the data. DC measures the overall strength of functional connectivity across the entire brain by computing the Pearson correlation coefficient between a voxel and all other voxels in the brain, followed by averaging the correlation coefficients of all voxels that exceed a threshold of 0.25. ReHo quantifies the degree of local synchronization by assessing the Kendall consistency coefficient between a voxel and its 26 nearest neighbors. Using PIQ as a covariate, the ComBat Harmonization is applied to remove site effects from multiple open-accessed data sets^3^ (Johnson et al., 2007; Reardon et al., 2021; Chen et al., 2022; Du et al., 2023). Finally, the two-sample *t*-test was then used for inter-group difference statistics, and the resulting map was calibrated by Gaussian random field correction (GRF, two-tailed voxel-level *p* < 0.01 and cluster-level *p* < 0.05) (Zhan et al., 2016; Gu et al., 2020; Wang et al., 2022).

#### Spatial matching of gene expression data

The whole-brain gene expression data in our work were obtained from the freely accessible Allen Human Brain Atlas (AHBA),^4^ which contained almost all the gene expression states in the brain regions in the Brodmann template. To refine and optimize the gene expression data, Abagen^5^ is then utilized. This involved several steps including: 1. re-annotate of the microarray expression matrix according to the data provided by Arnatkevicˇiūtė, and then removal of the gene probe of invalid Entrez-ID; 2. exclusion of the gene probes with lower expression intensity than the background noise; 3. selection of the probe with the highest Spearman rank correlation between the microarray expression of each probe and the RNA-seq expression data of the corresponding gene, in cases where a gene symbol is paired with multiple gene probes. As only two right-brain donors were available in the AHBA, the present study focused solely on the gene expression data of the left hemisphere of the brain.

### PLS analysis

In this study, PLS^6^ was utilized to identify weighted linear combinations of functional activity and gene expression in different brain regions. Specifically, gene expression profiles were employed as predictive variables, while the functional activity characteristics of specific brain regions served as response variables. To address the issue of spatial autocorrelation in the functional feature data, we used permutation analysis to randomly sort the data (*n* = 10,000) and test the statistical significance of the maximum explainable variance information across different regions. We then performed the same displacement analysis (*n* = 10,000) for weighted gene expression patterns to verify spatial similarity between gene expression patterns and brain functional features. Upon confirming the significant dimensions in the PLS analysis results, we utilized Bootstrapping method to perform random sampling on the gene weights of these dimensions and estimate the gene variability. By calculating the ratio of gene weight to the standard error of gene variability, we sorted the final score to obtain the genes significantly related to brain functional activities.

### Enrichment analysis

In order to characterize the functional properties of genes associated with heterogeneous brains of ASD at all age stages, a metaspace analysis^7^ was performed using the Gene Ontology (GO) identifiers of the top 5% of weighted genes identified in PLS analysis. A significance threshold of 0.05 was set for the enrichment results, which were subsequently corrected using the False Discovery Rate (FDR) approach.

## Results

### Demographic characteristics

A total of 396 male subjects were included in this study and divided into 4 age groups: children (stage 1, ASD:111, TD:88, age range 6 ~ 12), adolescents (stage 2, ASD:34, TD:33, age range 12 ~ 15), young-adult (stage 3, ASD:50, TD:61, age range 15 ~ 30), adult (stage 4, ASD:7, TD: 12, age range 30 ~ 56). There was no age difference among the subjects at each age stage, and detailed demographic characteristics were shown in Table 1.

**Table 1:** Demographic characteristics.

	Stage 1 (6 ~ 12 years)			Stage 2 (12 ~ 15 years)		
	ASD (*n* = 111)	TD (*n* = 88)	*p*-value	ASD (*n* = 34)	TD (*n* = 33)	*p*-value
Age (std)	9.15 (1.65)	9.48 (1.55)	0.15	13.3 (0.82)	13.3 (0.85)	0.96
PIQ	106.9 (18.8)	108.7 (14.8)	0.47	103.3 (16.5)	106.8 (14.5)	0.36
ADOS-total	12.0 (4.5)	/	/	13.8 (4.42)	/	/
SRS-total	90.4 (28.4)	/	/	97.6 (31.7)	/	/

	Stage 3 (15 ~ 30 years)			Stage 4 (>30 years)		
	ASD (*n* = 50)	TD (*n* = 61)	*p*-value	ASD (*n* = 7)	TD (*n* = 12)	*p*-value
Age (std)	20.2 (3.96)	19.8 (3.82)	0.60	35.6 (3.1)	37.5 (7.94)	0.55
PIQ	105.3 (15.9)	107.3 (13.9)	0.49	107.6 (15.7)	107.5 (8.66)	0.99
ADOS-total	12.4 (3.81)	/	/	13(4.73)	/	/
SRS-total	93.1 (29.8)	/	/	/	/	/

ASD, Autism Spectrum Disorder; TD, Typical development; PIQ, performance intelligence quotient; ADOS-total, Autism Diagnostic Observation Schedule—Total Scale; SRS-total, Social Responsiveness Scale—Total Scale. *p*-value was gained by the two-sample *t*-test.

### Heterogeneous brain region

Statistical analysis found that ASD showed different brain development trajectories compared with TD in all four age groups.

#### ALFF: ASD-TD

During stage 1, individuals with ASD showed increased ALFF in several brain regions, including the left orbitofrontal cortex (OFC_L), left inferior frontal gyrus (IFG_L), Broca area, subgenual cortex (SG_L), right fusiform gyrus (FFG_R), right superior temporal gyrus (STG_R), bilateral dorsal anterior cingulate cortex (dACC), anterior cingulate cortex (ACC), and perirhinal cortex. However, the bilateral primary visual cortex (V1) exhibited a decrease in ALFF. Stage 2 showed an increase in ALFF in the left orbitofrontal cortex (OFC_L), while a decrease was observed in the left fusiform gyrus (FFG_L) and bilateral ventral anterior cingulate cortex (vACC). In stage 3, ALFF was found to be increased in the left Broca’s area, insular cortex (IC_L), subcentral area (SCA_L), right frontal eye field (FEF_R), bilateral inferior frontal gyrus (IFG), and dorsolateral prefrontal cortex (dlPFC). Conversely, a decrease in ALFF was observed in the right inferior temporal gyrus (ITG_R), fusiform gyrus (FFG_R), temporal pole (TP_R), and primary auditory cortex (A1_R). Finally, stage 4 showed an increase in the right inferior temporal gyrus (ITG_R), fusiform gyrus (FFG_R), bilateral orbitofrontal cortex (OFC), and temporal pole (TP).

#### fALFF: ASD-TD

As per the findings of the study, changes were observed in the functional connectivity across various brain regions during different stages. The bilateral Broca area, subgenual cortex, perirhinal cortex, and medial temporal gyrus (MTG) exhibited an increase in activity during stage 1, whereas a decrease in activity was observed in the right fusiform gyrus (FFG_R) and bilateral dorsal posterior cingulate cortex (dPCC) and superior parietal lobule (SPL). In stage 2, the left frontal pole, anterior medial prefrontal cortex (amPFC_L), and right frontal pole showed an increase in activity, whereas the bilateral dorsal anterior cingulate cortex (dACC), orbitofrontal cortex (OFC), and anterior cingulate cortex (ACC) exhibited a decrease in activity. Stage 3, however, did not reveal any significant differences in functional connectivity. In stage 4, a decrease in functional connectivity was observed in the left primary motor cortex (M1_L), and somatosensory cortex (SSC_L), whereas an increase in activity was noted in the right temporal pole, inferior temporal gyrus (ITG_R), Broca area, and insular cortex, as determined by fALFF.

#### DC: ASD-TD

According to the results of the study, alterations in the DC were observed across different brain regions during various stages. In stage 1, an increase in DC was observed in the left visual association cortex (V2_L), bilateral inferior frontal gyrus (IFG), superior frontal gyrus (SFG), dorsolateral prefrontal cortex (dlPFC), frontal eye field (FEF), dorsal anterior cingulate cortex (dACC), and paraorhinal cortex, while a decrease in DC was observed in the bilateral orbitofrontal cortex (OFC), anteromedial prefrontal cortex (amPFC), dorsal posterior cingulate cortex (dPCC), visual association cortex (V2), and somatosensory association cortex (SAC). In stage 2, an increase in DC was observed in the bilateral dorsolateral prefrontal cortex (dlPFC), frontal eye field (FEF), somatosensory association cortex (SAC), and posterior central gyrus (PCG), whereas bilateral temporal polar regions, posterior cingulate cortex (PCC) and ventral anterior cingulate cortex (vACC) exhibited a decrease in DC. The bilateral thalamus demonstrated a reduction in DC in stage 3, while in stage 4, an increase in DC was observed in the bilateral orbitofrontal cortex (OFC), dorsal anterior cingulate cortex (dACC), superior frontal gyrus (SFG), temporal pole, inferior temporal gyrus (ITG), fusiform gyrus (FFG), inferior central gyrus (ICG), and dorsolateral prefrontal cortex (dlPFC). However, a reduction in DC was observed in the left somatosensory association cortex (SAC_L), the right fusiform gyrus (FFG_R), and bilateral primary visual cortex (V1), and visual association cortex (V2) during this stage.

#### ReHo: ASD-TD

**Figure 1:**
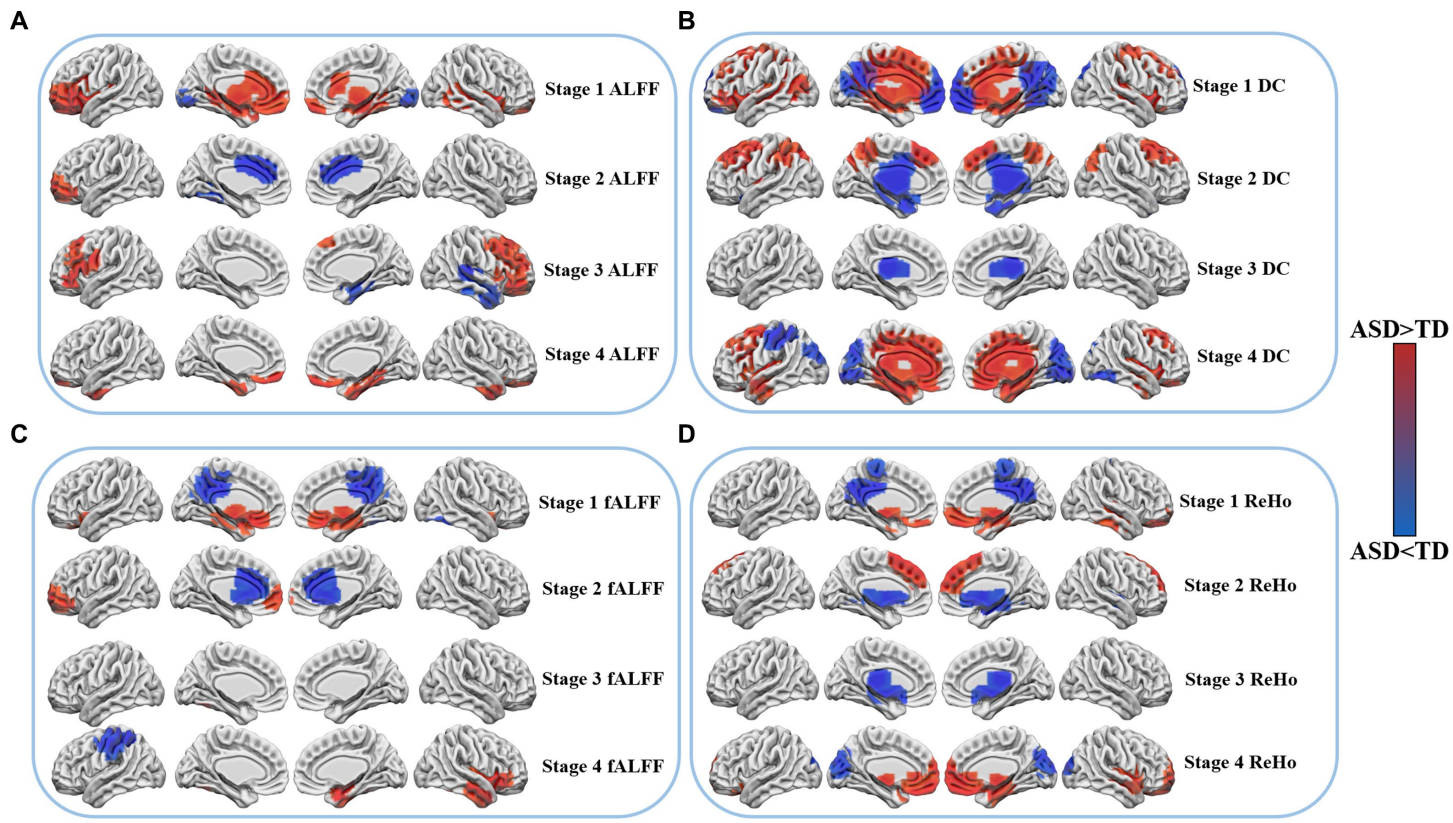
Differences in resting state fMRI between patients with ASD and TD at four age stages. Cool colors indicate lower functional activity of ASD than TD, while warm colors indicate higher functional activity of ASD than TD. **(A)** Shows the changes of ALFF in four age stages. **(B)** Shows the changes of DC in four age stages. **(C)** Shows the changes of fALFF in four age stages. **(D)** Shows the changes of ReHo in four age stages.

In stage 1, an increase in ReHo was observed in the right inferior temporal gyrus (ITG_R), bilateral subgenual cortex, medial temporal gyrus (MTG), and orbitofrontal cortex (OFC), while a decrease in ReHo was observed in the bilateral dorsal posterior cingulate cortex (dPCC), somatosensory association cortex (SAC), and ventral posterior cingulate cortex (vPCC). In stage 2, an increase in ReHo was observed in the bilateral dorsolateral prefrontal cortex (dlPFC) and frontal eye field (FEF), while a decrease in ReHo was observed in the bilateral perirhinal Cortex and cingulate cortex (CC). The bilateral thalamus and amygdala demonstrated a decrease in ReHo in stage 3. In stage 4, an increase in ReHo was observed in the right temporal polar (TP_R), inferior temporal gyrus cortex (ITG_R), bilateral orbitofrontal cortex (OFC), subgenual cortex, and medial temporal gyrus (MTG), whereas the ReHo of the bilateral primary visual cortex (V1) and visual association cortex (V2) exhibited a decrease, as illustrated in Figure 1.

### Enrichment analysis

There are a total of 58,692 gene probes in the gene data we got from the ALLEN BRAIN MAP, which corresponds to 29,180 original gene symbols. We kept 13,561 valid genes after re-annotating them. After the statistical analysis between the groups and the different brain regions was found, the expression profiles of significantly related genes were further enriched and analyzed. We found that the top 5% of PLS+ associated genes were involved in different functional enrichment analyses at four ages.

#### ALFF

During the first stage, the related genes of ALFF contribute significantly to the regulation of cell morphogenesis and modulation of chemical synaptic transmission. In the subsequent developmental stage, these genes assume an important role in protein localization and position maintenance on organelles. In the third stage, the genes are implicated in the response to oxygen content and protein localization on organelles. Furthermore, in the fourth stage, ALFF is found to be involved in the transmembrane transport activity of inorganic molecular entities, transmembrane transporter activity, and mitochondrial membrane.

#### fALFF

In the first stage, the associated genes of fALFF play a pivotal role in axon formation and intracellular protein transport. Moving ahead to the subsequent developmental stage, these genes are implicated in cell front, GTPase regulation activity, and cerebellar cortex development. Moreover, in the third stage, the genes are found to be associated with chromosome organization and DNA metabolism. Finally, in stage 4, fALFF is involved in monatomic cationic transport and catalytic activity that acts on nucleic acids.

#### DC

**Figure 2:**
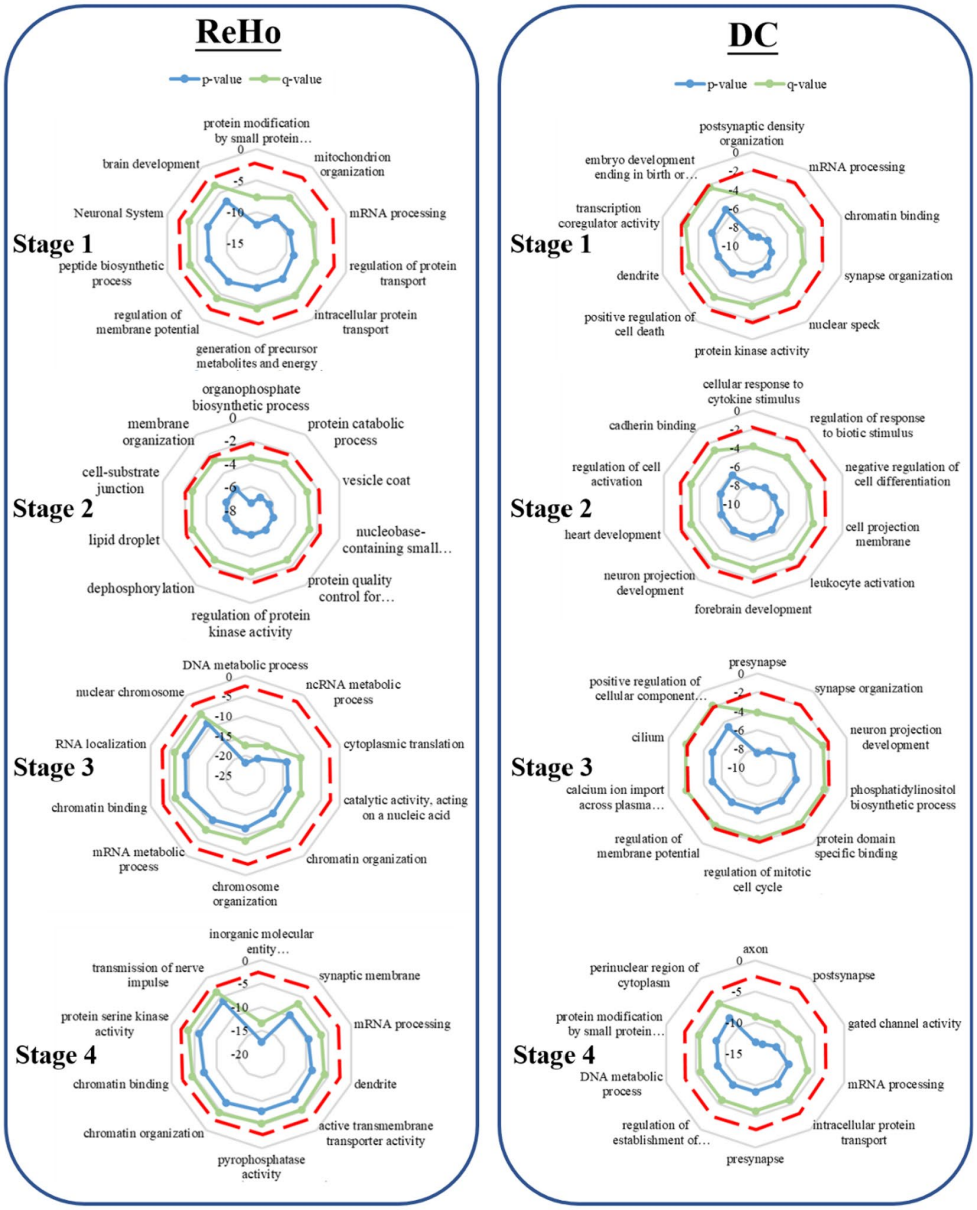
Gene enrichment analysis. The radar map represents the GO enrichment analysis of the weighted gene design identified by PLS. The red dotted line represents the significance threshold (*p* < 0.05), the green line represents the *p*-value after correction, and the blue line represents the *p*-value before correction.

**Figure 3:**
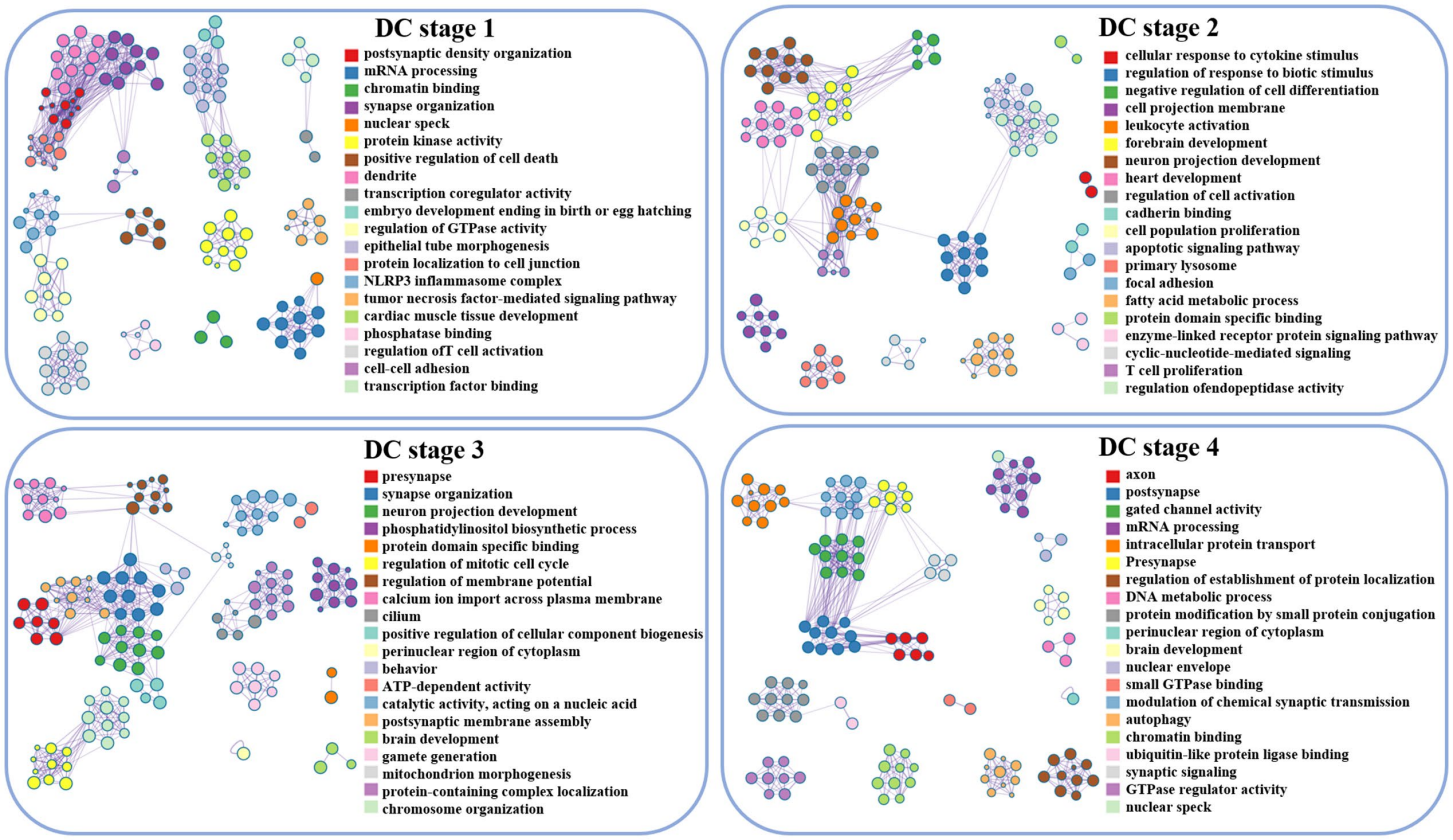
Visualization of DC enrichment analysis, lines between clusters indicate whether there is a correlation, and nodes with the same color belong to the same cluster. The term’s degree of significance increases with the darker the hue, and it decreases from top to bottom.

During the initial stage, the associated genes of DC are implicated in postsynaptic membrane density and mRNA processing. Subsequently, in the second developmental stage, these genes are involved in forebrain development and neuronal projection development in response to cytokine stimulation. Moreover, in the third stage, the genes are found to be associated with dendritic, synaptic organization, neuronal projection development, and membrane potential regulation. Finally, in stage 4, the activity of axons, dendrites, and gated channels is implicated (Figures 2, 3).

#### ReHo

**Figure 4:**
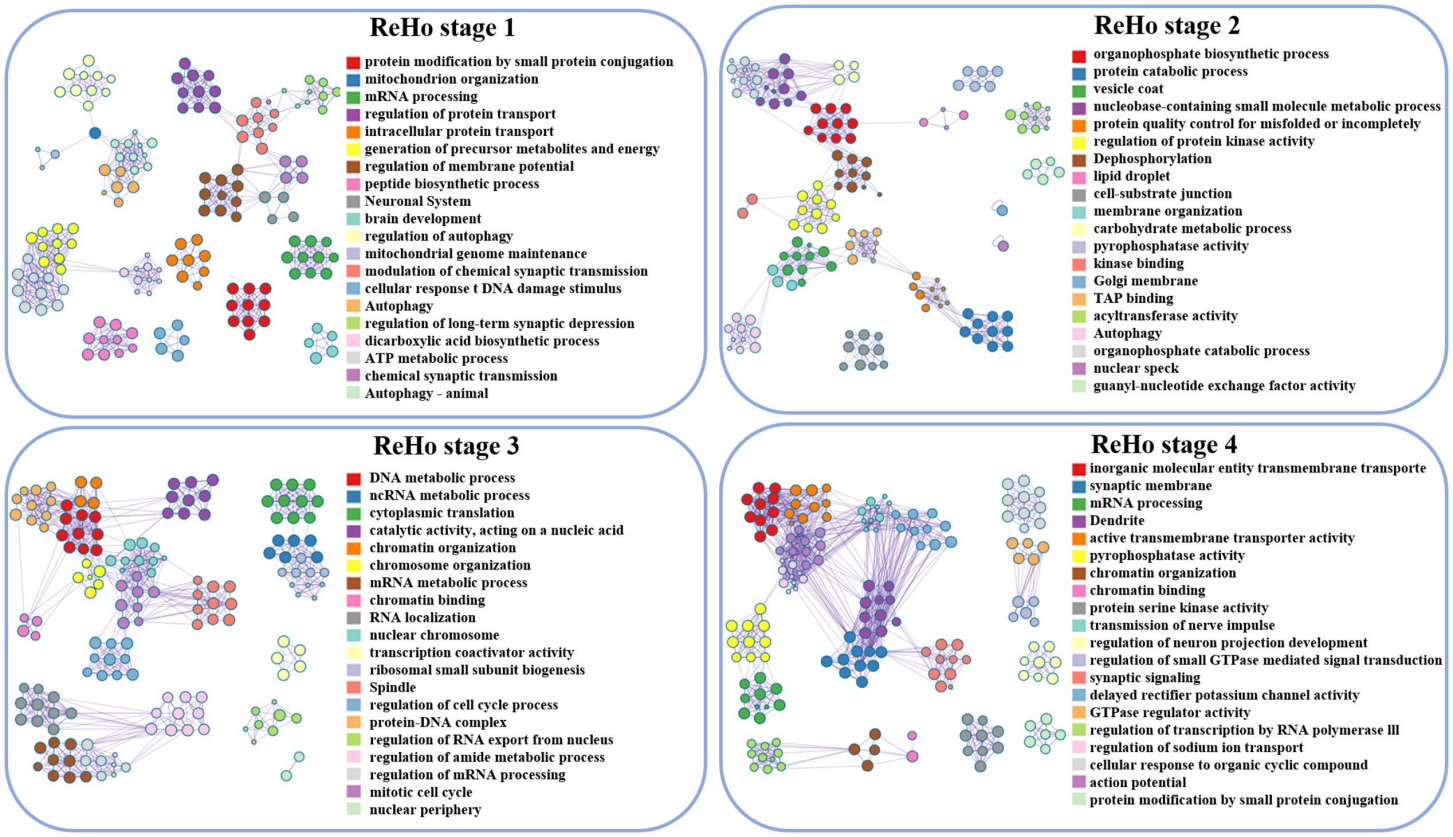
Visualization of ReHo enrichment analysis, lines between clusters indicate whether there is a correlation, and nodes with the same color belong to the same cluster. The term’s degree of significance increases with the darker the hue, and it decreases from top to bottom.

During the initial stage, the associated genes of ReHo play a significant role in protein modification by small protein conjugation, mitochondrial tissue, and membrane potential regulation. Moving ahead to the subsequent developmental stage, these genes are involved in organophosphorus biosynthesis, protein decomposition, and vesicle coat. Furthermore, in the third stage, the genes are associated with DNA metabolism and chromosome organization. Finally, in stage 4, ReHo is implicated in the activity of transmembrane transporters of inorganic molecular entities, synaptic membranes, and the transmission of nerve impulses, as depicted in (Figures 2, 4).

## Discussion

The present study aims to investigate the critical stages in brain development that demand attention, with a specific focus on ASD. According to the typical clinical manifestations of ASD, patients are divided into four age groups in this paper. The study aims to explore the abnormal alterations in cerebral cortex function and potential gene transcription during the life cycle of ASD by analyzing functional neuroimaging data and brain gene transcription profiles. The results of this study would provide a better understanding of the underlying pathophysiological mechanisms underlying ASD, and underscore the significance of considering developmental stages when investigating brain-related disorders (Figures 2, 4).

Functional activity analysis revealed that the prefrontal lobe, temporal lobe, and cingulate cortex exhibited distinctive changes throughout the life cycle of individuals with ASD. Additionally, the associated genes were found to be enriched in transmembrane transport activity of inorganic molecules, regulation of protein transport, organophosphorus biosynthesis, and DNA metabolism. These findings may provide a novel perspective to understand the atypical course of brain function development and possible biological anomalies underlying ASD.

Recent studies have revealed that male patients are more susceptible to ASD than female patients, with a ratio of 4.5:1 (Loomes et al., 2017). Furthermore, the differences in brain development patterns and biological changes between male and female patients cannot be overlooked during the preprocessing stage (Joel et al., 2015; Wang et al., 2021). In order to better understand these atypical developmental trajectory changes associated with ASD, it is essential to strictly control for gender differences. Thus, we only utilized resting-state fMRI data of male ASD and TD to avoid errors resulting from gender differences.

Throughout all four ages, our results show that the developmental trajectories of the frontal-temporal-cingulate cortex circuitry differ in the ASD brain. Notably, the frontal cortex, which plays a crucial role in cognitive, emotional, and behavioral control, presents heterogeneous changes across all four age groups (Badre and Nee, 2018). Specifically, the left frontal lobe ALFF variation region gradually declines with age, while the abnormal ALFF region shifts to the insular cortex in young adults (stage 3). By adulthood (stage 4), the ALFF abnormalities in the frontal lobe are minimized. However, the right dorsolateral frontal cortex, responsible for higher cognitive functions such as spatial perception, vision, and spatial memory (Kleinhans et al., 2008), shows the greatest variation in young adults (stage 3). As such, we underscore the importance of considering these developmental changes in the brain circuitry when investigating ASD.

In DC, we observed significant phased changes in the precuneus, cingulate cortex, ventral prefrontal cortex, and dorsomedial prefrontal cortex of DMN. The global functional connections of the precuneus decreased at stage 1 and increased at stage 2. Prior studies conducted on children with ASD have shown that the precuneus exhibited decreased connectivity with the visual cortex, basal ganglia, and local posterior medial cortex (Lynch et al., 2013). Similarly, a study conducted on young adults with ASD showed that functional connectivity in the precuneus and cingulate cortex with other DMN core regions was decreased, and this reduction was negatively associated with symptom severity (Assaf et al., 2010).

Significant phased changes were observed in the local functional connectivity similarity (ReHo) of the dorsomedial prefrontal cortex, precuneus, and right temporal lobe of the ventromedial prefrontal cortex. The temporal lobe is a crucial brain region in the cerebral cortex and plays an important role in various advanced cognitive functions, including hearing, memory, language understanding, visual recognition, and emotional processing (Carter and Huettel, 2013). It has been reported that the area of abnormal activation of the superior temporal sulci (STS) is highly correlated with ASD in adolescents and its grayscale density decreases significantly (Williams et al., 2006; Zilbovicius et al., 2006). Additionally, children with ASD exhibit higher activity in bilateral temporal lobes compared to the TD group, indicating difficulties in understanding the communication intentions of others (Wang et al., 2006). These findings provide insights into the atypical development of local functional connectivity similarity in the temporal lobe of individuals with ASD.

Existing studies have shown that individuals with ASD exhibit reduced volume of frontal lobe parenchyma and decreased white matter density compared to neurotypical controls (Schmitz et al., 2007). Furthermore, mixed-age studies have found that individuals with ASD exhibit increased activation in the right frontal lobe and right upper temporal lobe compared to control groups (Kleinhans et al., 2008). Our findings are consistent with these studies but also reveal a non-linear temporal lobe functional activity pattern, characterized by increased activity in children (stage 1), decreased activity in young adults (stage 3), and increased activity in adults (stage 4). This non-linearity suggests that age-related changes in aberrant brain areas may not follow a linear progression. Notably, our study also identifies non-linear changes in ReHo and DC, which may represent a distinct developmental trajectory unique to ASD.

The expression of specific genes in the human genome is closely related to the brain. It is widely believed that many psychiatric diseases are caused by the intricate interplay of multiple genes, the proteins encoded by these genes may play important regulatory roles in various brain processes, such as neuronal development, interneuronal signal transmission, neural plasticity, etc. (Wu et al., 2015). If these genes are mutated or abnormally expressed, they may lead to neuron damage, neural pathway disorders, and eventually psychiatric diseases. Therefore, it is crucial to examine the relationship between neuroimaging and the genetic transcription in ASD to better understand the underlying mechanisms of the disorder.

The brain’s development and function are significantly affected by proteins, RNA, and other functional molecules encoded by genes. In cortical cells, the activation or inhibition of different genes produces different proteins, which may influence the shape, function, and connection of neurons, thereby modulating different aspects of cognitive, emotional, and behavioral brain functions (Auid et al., 2018).

In this study, we employed PLS to detect genes linked with regions exhibiting atypical brain function in ASD and subsequently conducted a functional enrichment analysis of these genes. In general, these genes are mainly related to synaptic organization, protein transport regulation, neuronal projection development, and DNA metabolism. ASD is a typical neurodevelopmental disorder characterized by complex and heterogeneous etiology and pathogenesis. Notwithstanding the uncertainty in the field, existing literature suggests that abnormal brain neuron function could play a pivotal role in the onset of ASD (Bernier et al., 2014).

Our analysis revealed that genes associated with resting-state functional connectivity metrics, such as ReHo and DC, were significantly enriched in terms related to mRNA processing during stage 1 and stage 4 of brain development. Interestingly, several studies have demonstrated that mutations in specific genes or abnormal expression of mRNA can impair the function, development, and signal transduction of neurons, which has been implicated in ASD pathology (Bagni and Zukin, 2019). Studies have also shown that it is related to cognition. Furthermore, the concentration of the emotion-associated dopamine receptor D4 (DRD4-mRNA) was significantly reduced in children and adolescents with ASD (Taurines et al., 2011). It is worth mentioning that the synaptic structure of neuron cells was found to be significantly enriched with genes associated with global functional connectivity (DC) during stages 1, 3, and 4. The release of neurotransmitters to alter the potential of receiving neurons or trigger physiological responses is the primary purpose of synapses. The NRXN, NLGN, and SHANK genes are mutated most frequently at synapses, according to several studies. Thus, it is possible that synaptic disruption plays a key role in the etiology of ASD (Gai et al., 2012; Guang et al., 2018). These findings demonstrate that genes associated with abnormal brain regions are involved in and influence neuronal dysfunction across the lifespan of ASD, which could ultimately lead to abnormal neural function in ASD individuals. In addition, we found that genes associated with different resting-state functional connectivity metrics were enriched in distinct biological processes during different stages of brain development. Specifically, the associated genes identified in stage 2 using the four calculation methods of ALFF, hair ALFF, DC, and ReHo were mainly enriched in terms such as molecular metabolism, regulation, and differentiation of cells. The genes associated with ALFF, fALFF, and ReHo in stage 4 are all involved in the transmembrane transport of ions between cells. Although the results in DC do not involve this process, the activity of gated channels is regulated by the transmembrane transport of ions (Randak and Welsh, 2003; Hwang and Kirk, 2013). Voltage-controlled calcium channels, which are regulated by the transmembrane transport of ions, play a crucial role in dendritic growth and arborization of neuronal cells (Krey et al., 2013). However, knocking out genes encoding voltage-gated channels in mice did not lead to neurological symptoms (Pinggera and Striessnig, 2016). Collectively, our findings suggest that genes associated with different resting-state functional connectivity metrics could contribute to distinct biological processes, which could potentially be targeted for the development of novel ASD therapeutics.

## Limitation

The present study has several limitations that should be taken into consideration. Firstly, the age divisions utilized in our analysis were limited and may not fully capture the intricacies of the developmental trajectory of ASD. To gain a more nuanced understanding of the progression of autism, future research could benefit from utilizing more refined age categories or collecting longitudinal data.

## Data availability statement

The original contributions presented in the study are included in the article/Supplementary material, further inquiries can be directed to the corresponding author.

## Ethics statement

The data used in this study is obtained from the open-source dataset from ABIDE. The ethical considerations pertaining to human participants in this study were meticulously examined and approved by the Institutional Review Board. Written informed consent was obtained from all patients/participants prior to their involvement in the study. For instance, The studies involving human participants were reviewed and approved by Ethical approval was obtained from the St. James’s Hospital/AMNCH (ref: 2010/09/07) and the Linn Dara CAMHS Ethics Committees (ref: 2010/12/07). Written informed consent to participate in this study was provided by the participants’ legal guardian/next of kin.

## Author contributions

To ensure the experiments’ progress and caliber, YL created the research plan, planned the experimental procedure, and was in charge of the majority of the experiments. FZ oversaw the data gathering, experimental collation, in-depth analysis of the findings, and creation of the manuscript. RL oversaw organizing the background information and the border of connected technical domains in the manuscript and presents some fresh theoretical stances and concepts. JG and JH assisted in the revision of the work and edited the expression and structure to make it more quality and readable. All authors contributed to the article and approved the submitted version.

## Conflict of interest

The authors declare that the research was conducted in the absence of any commercial or financial relationships that could be construed as a potential conflict of interest.

## Publisher’s note

All claims expressed in this article are solely those of the authors and do not necessarily represent those of their affiliated organizations, or those of the publisher, the editors and the reviewers. Any product that may be evaluated in this article, or claim that may be made by its manufacturer, is not guaranteed or endorsed by the publisher.

## Footnotes

^1^ http://fcon_1000.projects.nitrc.org/indi/abide

^2^ http://www.rfmri.org/DPARSF

^3^ https://github.com/Jfortin1/ComBatHarmonization

^4^ http://human.brain-map.org

^5^ https://abagen.readthedocs.io/en/stable/

^6^ https://github.com/helab207/Alterations-in-Connectome-Dynamics-in-ASD-main

^7^ http://metascape.org/gp/index.html#/main/step1
