# Supplementary material for: Exploring the correlation between genetic transcription and multi-temporal developmental autism spectrum disorder using resting-state functional magnetic resonance imaging
Source: Front Neurosci. 2023 Jun 29;17:1219753. doi: 10.3389/fnins.2023.1219753 (PMC10339831; doi:10.3389/fnins.2023.1219753)
Supplement: Supplementary file 1 [file Table_1.DOCX]

*Supplementary Material*

Exploring the correlation between genetic transcription and multi-temporal developmental autism spectrum disorder using resting-state functional magnetic resonance imaging

Yanling Li^1*^, Fanchao Zhou^1^, Rui Li^1^ Jiahe Gu^1^, Jiangping He^1^

^1^ School of Electrical Engineering and Electronic Information, Xihua University, Chengdu, 610039, China

*** Correspondence:**Dr. Yanling Li

153275236@qq.com

| ABIDE | SITE | scanner manufactured | number of slice | fMRI scan length (min:sec) | fMRI scan resolution (mm) | fMRI TR (ms) | Number of Measurements | FoV(mm2) | Acquisition Matrix |
| --- | --- | --- | --- | --- | --- | --- | --- | --- | --- |
| 1 | PITT | Siemens | 29 | 5:06 | 3.1*3.1*4.0 | 1500 | 200 | 200*200 | 64*64 |
| 1 | SU | GE | 31 | unavailable | 3.4375*3.4375*3.5 | 2000 | 180 | 220*220 | 64*64 |
| 1 | NYU | Siemens | 33 | 6:00 | 3.0*3.0*4.0 | 2000 | 180 | 240*240 | 80*80 |
| 1 | CALTECH | Siemens | 34 | 5:04 | 3.5*3.5*3.5 | 2000 | 150 | 224*224 | 64*64 |
| 1 | YALE | Siemens | 34 | 6:40 | 3.4*3.4*4.0 | 2000 | 200 | 240*240 | 80*80 |
| 2 | SDSU | GE | 42 | 6:10 | 3.4375*3.4375*3.4 | 2000 | 180 | 220*220 | 64*64 |
| 2 | NYU | Siemens | 33 | 6:00 | 3.0*3.0*4.0 | 2000 | 180 | 240*240 | 80*80 |
| 2 | EMC | GE | 37 | 5:20 | 3.6*3.6*4.0 | 2000 | 160 | 230*230 | 64*64 |

1. **Supplementary Figures and Tables**

Table S1. **Description of the scanning parameters.** PITT: University of Pittsburgh, SU: Stanford University, NYU: New York University Langone Medical Center, CALTECH: California Institute of Technology, YALE: Yale University, SDSU: San Diego State University, EMC: Erasmus University Medical Center.
